# Bithyniid snails (Gastropoda: Bithyniidae) infected with Xiphidiocercariae in Thailand include a new record of *Bithynia siamensis siamensis* as the intermediate host of *Plagiorchis* and *Paralecithodendrium*

**DOI:** 10.1371/journal.pone.0317052

**Published:** 2025-02-04

**Authors:** Abdulhakam Dumidae, Jiranun Ardpairin, Supawan Pansri, Chanatinat Homkaew, Mayura Nichitcharoen, Aunchalee Thanwisai, Apichat Vitta

**Affiliations:** 1 Faculty of Medical Science, Department of Microbiology and Parasitology, Naresuan University, Phitsanulok, Thailand; 2 Faculty of Medical Science, Centre of Excellence in Medical Biotechnology (CEMB), Naresuan University, Phitsanulok, Thailand; 3 Faculty of Sciences, Center of Excellence for Biodiversity, Naresuan University, Phitsanulok, Thailand; Universidade Federal de Minas Gerais, BRAZIL

## Abstract

Bithyniids are freshwater snails that play a crucial role in the transmission of various parasitic trematodes of medical and veterinary importance. In this study, we explored the prevalence of cercarial trematode infections in bithyniid snails from Thailand and examined the species diversity of both the intermediate snail hosts and parasite larvae. A total of 688 bithyniid snails were collected from diverse natural habitats at 24 locations in 16 provinces across 5 regions of Thailand. The presence of larval trematode infections was examined using the cercarial shedding method. Both the collected snails and the emerging cercariae were identified at the species level using a combination of morphological and molecular techniques. The mitochondrial COI and 16S rDNA sequences of bithyniid snails, along with the ITS2 sequences of cercariae, were obtained via PCR amplification and sequencing. Three species of bithyniid snails were identified in this study: *Bithynia funiculata*, *Bithynia siamensis siamensi*s, and *Hydrobioides nassa*. Among these species, *B*. *s*. *siamensis* exhibited the highest population density, followed by *B*. *funiculata* and *H*. *nassa*. The overall rate of cercarial infection in the bithyniid snails was relatively low, at 1.45%. *H*. *nassa* snails had the highest infection prevalence, at 11.11%, while *B*. *s*. *siamensis* had a prevalence of 1.39%. Only the morphological type of the xiphidiocercariae was detected. BLASTn searches in GenBank and phylogenetic trees based on xiphidiocercariae were used to classify the samples into four different families spanning two superfamilies of digenean trematodes. The genera *Plagiorchis*, *Prosthogonimus*, *Paralecithodendrium*, and cercaria of Renicolidae are reported for the first time in *B*. *s*. *siamensis*. *Plagiorchis* and *Paralecithodendrium* are significant genera of zoonotic trematodes. These findings indicate that *B*. *s*. *siamensis* and *H*. *nassa* can act as the first intermediate hosts for various parasitic trematodes in Thailand.

## Introduction

Digenean trematodes, also known as flukes, are a group of parasitic flatworms that are significant parasites of humans and domestic animals, leading to numerous public health and livestock issues [1,2]. Many trematodes, particularly food-borne trematodes, have been documented to cause diseases in humans across numerous countries worldwide [3]. Foodborne trematodiases are a major group of neglected tropical diseases, with more than 40 million people infected and more than 750 million at risk (>10% of the world’s population) [4,5]. Furthermore, more than 100 species of foodborne trematodes are known to infect humans [3]. In Southeast Asia, humans are exposed to at least 70 species of foodborne and waterborne trematodes, which include lung flukes, liver flukes, intestinal flukes, and blood flukes [6]. These infections typically occur in focal outbreaks and remain endemic in various regions worldwide, notably in Southeast Asia, including Thailand [3,7].

Freshwater snails play a crucial role as intermediate hosts for numerous trematode species [8]. In general, the life cycle of trematodes typically involves freshwater and marine snails as the intermediate host [9]. After egg hatching, the miracidium stage is released, initiating larval development within a suitable snail host. The larvae undergo asexual multiplication, producing many cercariae [10]. The cercariae larvae emerge from the first intermediate host snail and penetrate the second intermediate host. Within the second intermediate host, the cercariae encyst and develop into the infective metacercariae stage. Humans or animals ingest metacercariae as definitive hosts, allowing them to develop into adults and complete their life cycle [10]. Most trematodes typically utilize specific species of freshwater snails as their first intermediate hosts [11]. Therefore, the circulation of trematodes relies on the presence of intermediate hosts [12]. Various snail species, particularly those belonging to the family Bithyniidae, are known to be the first intermediate hosts for medically significant parasitic trematodes endemic to Thailand [13].

Freshwater snails of the family Bithyniidae were first documented in Europe and Asia in the early 1870s [14,15]. In Thailand, ten species and subspecies have been identified and are distributed across four genera: *Bithynia*, *Hydrobioides*, *Wattebledia*, and *Gabbia* [16]. Previous research has shown that bithyniid snails, particularly those in the genera *Bithynia* and *Hydrobioides*, act as the first intermediate hosts for several medically and veterinary important trematodes. These include plagiorchiids, heterophyids, lecithodendriids, echinostomes, gastrothylacids, schistosomes, and the carcinogenic liver fluke *Opisthorchis viverrini*, which pose serious threats to public health [1,13,17]. Currently, in Thailand, the genus *Bithynia* is classified into two species: *Bithynia siamensis* and *B*. *funiculata*. *Bithynia siamensis* is further categorized into two subspecies, *B*. *s*. *siamensis* and *B*. *s*. *goniomphalos*. However, the morphology of *B*. *s*. *siamensis* and *B*. *s*. *goniomphalos* resembles that of *Hydrobioides nassa*, leading to confusion when traditional morphological characters are used for identification and classification [18]. Therefore, molecular methods have been used for species identification in previous studies. Specifically, mitochondrial genes such as cytochrome c oxidase subunit I (COI) and 16S rDNA have been utilized to distinguish between *Bithynia* and *Hydrobioides* snails, as demonstrated by Kulsantiwong et al. (2013) [19] and Bunchom et al. (2021) [18,20].

However, despite the crucial role of bithyniid snails in the life cycle of trematodes, there have been relatively few reports about trematode infections in these snails. For over a decade, there have been no recent reports on the infection rate and classification of trematode cercariae from bithyniid snails across various regions of Thailand. The most recent research dates from 2008–2009, when Kulsantiwong et al. (2015) [21] conducted a survey on trematode infections in freshwater snails of the family Bithyniidae in Thailand. The reported infection rate was 3.15%, and they identified six morphological types of cercariae. Additionally, a study by Kiatsopit et al. (2016) [22], in which snails were collected from 2009–2014, revealed that 20 morphologically distinct types of cercariae were identified in *B*. *s*. *goniomphalos* from the northeast regions. Most recently, in 2019, Tapdara et al. (2022) [17] collected *H*. *nassa* from three regions (north, west, and central) of Thailand and reported a prevalence of cercarial infection of 5.57%, with five different morphological types detected. However, the infection status of cercariae trematodes in bithyniid snails has not yet been assessed in many provinces in Thailand.

The conventional method used to identify trematode cercariae relies on their morphological characteristics, which can be quite challenging due to morphological similarities. This method typically allows identification only at the superfamily or family level [8]. However, identifying cercariae at the genus and species levels is often difficult or even impossible [23]. Correctly identifying cercariae at the genus and species levels is essential. Molecular genetic techniques offer higher resolution for identifying trematodes in their larval stage. Sequences from the nuclear ribosomal internal transcribed spacer 2 (ITS2) region have been utilized for identifying various stages, including the cercariae, metacercariae, and adult stages, in both intermediate and definitive hosts. They are also used in phylogenetic relationship analyses [24,25].

Trematode infectious diseases are predominantly endemic in areas where intermediate snail hosts are distributed [26]. However, the world is currently facing issues with rapidly growing snail populations, particularly near lakes and dams, leading to the widespread transmission of parasitic diseases [27,28]. As a result, current studies on trematode larval stages in snail hosts have garnered increasing attention because data on cercarial infection in snails are essential for developing effective strategies for the prevention and control of trematode diseases [29]. We hypothesize that bithyniid snails may be infected with diverse groups of zoonotic trematode parasites. Therefore, this study aimed to assess the prevalence of cercarial trematode infections in bithyniid snails from Thailand and to explore the species diversity of both intermediate snail hosts and parasite larvae.

## Materials and methods

### Snail collection and identification

**Fig 1:**
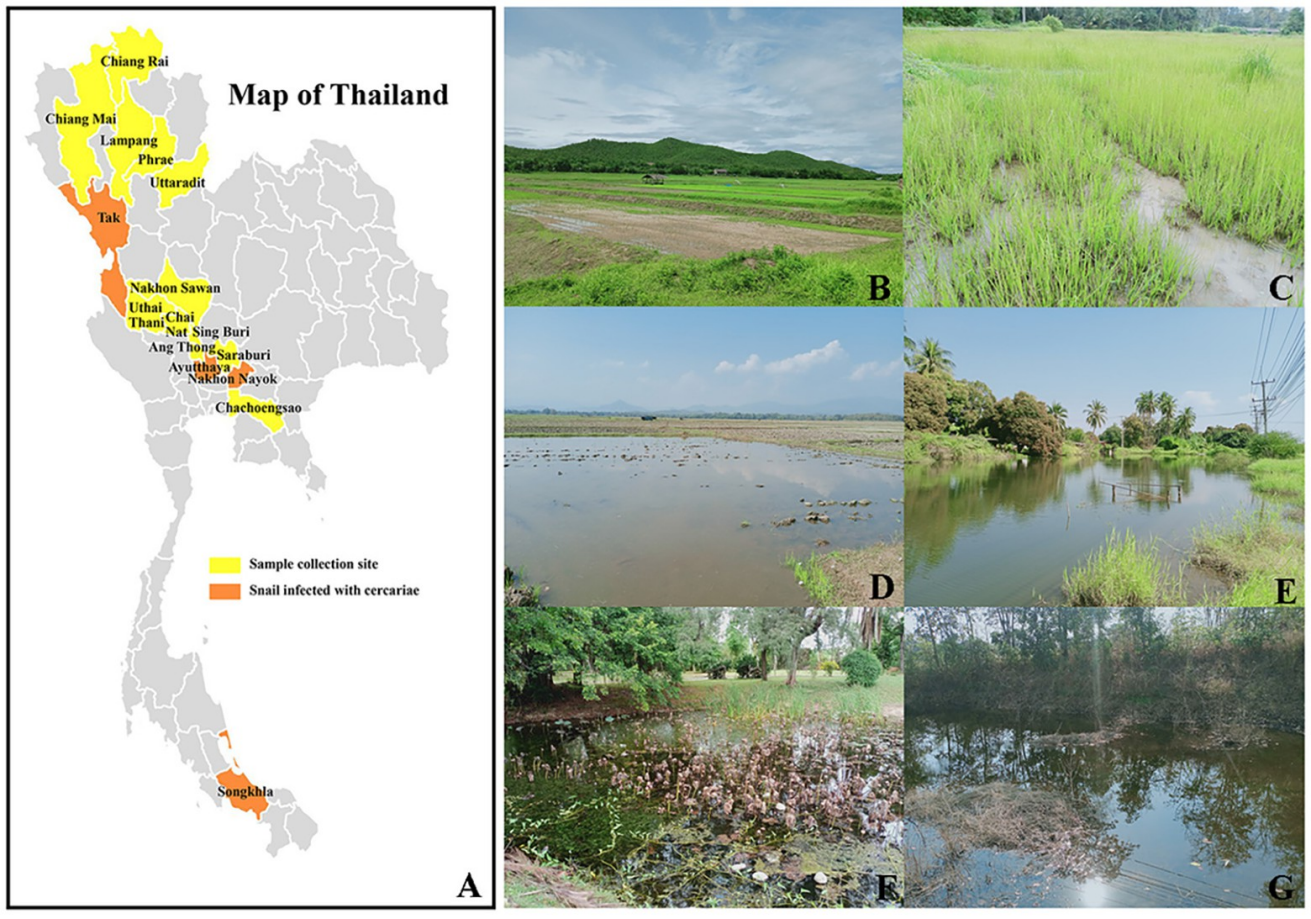
Geographic locations (A) and environments of the bithyniid snail collection sites examined in this study, including paddy fields (B-D), natural canals (E), lotus ponds (F), and irrigation canals (G). The map was created using MapChart software’s free version (https://www.mapchart.net/terms.html#licensing-maps), under a CC BY license, with permission from Minas Giannekas (owner and creator of the map-making website mapchart.net).

Bithyniid snails were randomly collected from 16 provinces across 5 geographical regions (central, eastern, northern, southern, and western) of Thailand (Fig 1 and Table 1). The samples were collected from their natural habitats, including paddy fields, canals, and ponds, using handpicking and scooping methods. The collected snails were placed in porous plastic bags with water and transported to the Department of Microbiology and Parasitology, Faculty of Medical Science, Naresuan University, Phitsanulok, Thailand. All snails were cleaned by washing with dechlorinated water and identified according to the standard morphological criteria of Brandt (1974) [14], Chitramvong (1992) [16], Bunchom et al. (2021) [18], and Kulsantiwong et al. (2022) [30]. Briefly, *B*. *s*. *siamensis* has a slender body shape, a narrow umbilicus, and a weak carina. The shell of *B*. *funiculata* is slightly larger, subovate-conic in shape, and varies in color from dull green to reddish-brown or olive-brown. It features a funnel-shaped umbilicus and a strong carina. The shell of *H*. *nassa* is small with transverse growth lines and spiral markings on its surface. The umbilicus is completely closed, while the outer part of the last whorl extends as a sinuous flange. The shell aperture has a slight incision at the middle of the basal lip. Additionally, molecular confirmation was conducted using mitochondrial DNA sequencing. The experiments involving invertebrate animals (snails) were approved by the Center for Animal Research at Naresuan University (Project Ethics No: NU-AQ640803).

**Table 1:** Sampling sites of bithyniid snails in this study.

Sampling site	Code	Latitude/Longitude	Region	Habitat types	No. of snails examined	*Bithynia funiculata*		*Bithynia siamensis siamensis*		*Hydrobioides nassa*		Total prevalence (%)
						No. of snails	No. of infected	No. of snails	No. of infected	No. of snails	No. of infected	
Chorakhe Rong, Chaiyo District, Ang Thong Province	ATG1	14.6493/100.4764	Central	Paddy field	40	0	0	40	0	0	0	0
Ban Len, Bang Pa-in District, Ayutthaya Province	AYA1	14.2282/100.6114	Central	Paddy field	210	0	0	210	1	0	0	1 (0.48)
Tha Chanuan, Manorom District, Chai Nat Province	CNT2	15.3413/100.1677	Central	Paddy field	22	0	0	22	0	0	0	0
Hang Nam Sakhon, Manorom District, Chai Nat Province	CNT3	15.3111/100.1842	Central	Canal	29	0	0	29	0	0	0	0
Nam Song, Phayuha Khiri District, Nakhon Sawan Province	NSN1	15.4261/100.1180	Central	Canal	20	0	0	20	0	0	0	0
Nong Krot, Banphot Phisai District, Nakhon Sawan Province	NSN2	16.0211/100.1140	Central	Paddy field	22	0	0	22	0	0	0	0
Thonglang, Ban Na District, Nakhon Nayok Province	NYK1	14.1862/101.0387	Central	Paddy field	33	0	0	33	5	0	0	5 (15.15)
Pa Kha, Ban Na District, Nakhon Nayok Province	NYK2	14.2801/101.0501	Central	Lotus pond	4	0	0	4	0	0	0	0
Chi Nam Rai, In Buri District, Sing Buri Province	SBR1	15.0609/100.3247	Central	Paddy field	83	0	0	81	0	2	0	0
	SBR2	15.0764/100.3115		Canal	53	0	0	52	0	1	0	0
Namtan, In Buri District, Sing Buri Province	SBR5	14.9625/100.3717	Central	Paddy field	10	0	0	10	0	0	0	0
Phu Khae, Chaloem Phra Kiat District, Saraburi Province	SRI1	14.6728/100.8850	Central	Irrigation canal	2	0	0	2	0	0	0	0
Hat Thanong, Mueang Uthai Thani District, Uthai Thani Province	UTI1	15.4198/100.0977	Central	Paddy field	1	0	0	1	0	0	0	0
Don Ko Ka, Bang Nam Priao District, Chachoengsao Province	CCO2	13.9407/101.0551	Eastern	Paddy field	20	0	0	20	0	0	0	0
Nong Ha, San Sai District, Chiang Mai Province	CMI1	18.8938/98.9944	Northern	Paddy field	4	4	0	0	0	0	0	0
Mueang Kaeo, Mae Rim District, Chiang Mai Province	CMI2	18.8882/98.9855	Northern	Paddy field	5	0	0	5	0	0	0	0
Pa Ko Dam, Mae Lao District, Chiang Rai Province	CRI1	19.7681/99.7369	Northern	Paddy field	23	23	0	0	0	0	0	0
Mae Tha, Mae Tha District, Lampang Province	LPG1	18.1725/99.5615	Northern	Paddy field	1	1	0	0	0	0	0	0
Mae Khammi, Mueang Phrae District, Phrae Province	PRE1	18.2538/100.2300	Northern	Paddy field	5	3	0	2	0	0	0	0
Nam Ang, Tron District, Uttaradit Province	UTT2	17.4576/100.2178	Northern	Paddy field	9	1	0	8	0	0	0	0
Phawong, Mueang Songkhla District, Songkhla Province	SKA2	7.1542/100.5762	Southern	Lotus pond	17	0	0	17	3	0	0	3 (17.66)
Nam Ruem, Mueang Tak District, Tak Province	TAK1	16.8900/99.2211	Western	Canal	7	1	0	5	0	1	1	1 (14.29)
Pa Mamuang, Mueang Tak District, Tak Province	TAK3	16.8671/99.1131	Western	Paddy field	34	0	0	29	0	5	0	0
Wang Prachop, Muang Tak District, Tak Province	TAK5	16.9145/99.3335	Western	Paddy field	34	0	0	34	0	0	0	0
**Total**					**688**	**33**	**0**	**646**	**9**	**9**	**1**	**10 (1.45)**

### Examination of cercaria infection

Trematode infection in bithyniid snails was assessed using the cercarial shedding technique [31]. Each snail was placed in a small plastic container (4 cm in diameter and 6.5 cm in height) filled with 20 ml of dechlorinated water. The container was covered with a small, perforated plastic lid to provide air ventilation and prevent the snails from escaping. The snails were exposed to natural light for 3–5 h during the day and kept at room temperature for 24 h [32]. Afterward, each container was daily examined for the presence of cercariae under a stereomicroscope for 7–10 days. The numbers of examined and infected snails were recorded to calculate the prevalence of infection. The living cercariae were primarily classified into types based on their morphological characteristics using light microscopy, according to standard identification methods [1,33]. Cercariae were photographed using an Olympus DP72 digital camera fitted to an Olympus BX53 microscope (Olympus Corporation, Japan). The cercariae were collected in a 1.5 ml sterile microcentrifuge tube and preserved at -20°C for DNA extraction. Afterward, the body of each snail that tested positive for cercariae was removed from its shell, sectioned into small pieces of body tissue (approximately 25 mg), and stored at -20°C for subsequent DNA analysis.

### DNA extraction

Genomic DNA from cercariae and snail samples was extracted using a NucleoSpin® Tissue Kit (Macherey-Nagel, Duren, Germany) following the manufacturer’s protocol. The DNA was eluted with 30 μl of elution buffer for cercariae and 70 μl for snail samples. The DNA concentration was assessed using 0.8% agarose gel electrophoresis in Tris-Boric-EDTA (TBE) buffer at 100 V, and the DNA was stained with ethidium bromide. The genomic DNA was then stored at -20°C until use for PCR analysis.

### Polymerase chain reaction (PCR) amplification and DNA sequencing

Cercariae DNA was amplified for the ITS2 region [34], while bithyniid DNA was amplified for COI [35] and 16S rDNA [36]. PCR amplification was performed in a total volume of 30 μl, consisting of 15 μl of OnePCR Ultra (Biohelix, New Taipei, Taiwan), 1.5 μl of each primer at 5 μM (0.25 μM), 9 μl of distilled water, and 3 μl of the extracted DNA template (20–200 ng). The amplified products were separated by gel electrophoresis at 100 V for 35 min using a 1.2% agarose gel stained with ethidium bromide. Subsequently, the PCR products were purified using the NucleoSpin® Gel and PCR Clean-Up Kit (Macherey-Nagel, Germany) according to the manufacturer’s instructions. The purified PCR products were then analyzed using a 1.2% agarose gel. Nucleotide sequencing was conducted by Macrogen, Korea. The details of the primers and PCR conditions used for the cercariae and snails used in this study are provided in Table 2.

**Table 2:** Primer sequences and PCR conditions used for amplifying trematode cercariae and bithyniid snails.

Gene or region	Primer sequence/(Reference)	PCR (bp)	PCR condition
COI	LCO1490_forward 5’-GGTCAACAAATCATAAAGATATTGG-3’ HCO2198_reverse 5’-TAAACTTCAGGGTGACCAAAAAATCA-3’ [35]	710	94°C/4 min; 94°C/1 min, 50–52°C/1 min, 72°C/1 min, 35 cycles; 72°C/8 min
16S rDNA	16Sar_forward 5’-CGCCTGTTTATCAAAAACAT-3’ 16Sbr_reverse 5’-CCGGTCTGAACTCAGATCACGT-3’ [36]	500	
ITS2	ITS3_forward 5′-GCATCGATGAAGAACGCAGC-3′ ITS4_reverse 5′-TCCTCCGCTTATTGATATGC-3′ [34]	480	94°C/5 min; 94°C/1 min, 56°C/1 min, 72°C/30 sec, 35 cycles; 72°C/10 min

### Molecular identification and phylogenetic analysis

The forward and reverse DNA sequence chromatograms were manually edited and assembled using SeqMan II (DNASTAR, Madison, WI, USA). The nucleotide sequences were subjected to BLASTn searches to identify similarities with sequences from other taxa in the GenBank database (National Center for Biotechnology Information: NCBI). The partial sequences obtained in the study, along with similar sequences selected from GenBank, were aligned using CLUSTAL W and trimmed with MEGA version 7.0 [37].

The sequence data from both the trematode ITS2 sequences and the snail COI and 16S rDNA mtDNA sequences generated in this study, as well as sequences from related reference species retrieved from the GenBank database, were used to construct phylogenetic trees. Phylogenetic relationships were analyzed using both maximum likelihood (ML) and neighbor-joining (NJ) methods. ML phylogenetic trees were constructed using the Tamura 3-parameter model [38] for 16S rDNA and ITS2 sequences and the Tamura-Nei model [39] for COI sequences. Moreover, NJ trees were generated utilizing the Kimura 2-parameter model [40] with bootstrap support of 1,000 replications using the MEGA version 7.0 program. Though two methods were used to establish the phylogeny, only the ML tree was selected for presentation in this study because both methods revealed congruent topologies. Additionally, genetic distances of the species were estimated using MEGA version 7.0 software to assess and compare genetic variation.

## Results

### Cercarial infection in snails

In this study, a total of 688 bithyniid snails were collected from 24 locations in 16 provinces across 5 regions of Thailand. Based on morphological identification, the snail samples belonged to three species: *Bithynia funiculata*, *Bithynia siamensis siamensis*, and *Hydrobioides nassa*. *B*. *s*. *siamensis* was the most abundant species, accounting for 93.89% (646/688) of the samples, followed by *B*. *funiculata* at 4.79% (33/688) and *H*. *nassa* at 1.31% (9/688). *B*. *s*. *siamensis* was found across all five regions (central, eastern, northern, southern, and western). In contrast, *B*. *funiculata* was limited to the northern and western regions, and *H*. *nassa* was found only in the central and western regions.

Out of the 688 bithyniid snails, ten were found to be infected with trematode cercariae and were distributed across 4 locations in 4 provinces of Thailand, resulting in an overall prevalence of infection of 1.45%. *H*. *nassa* exhibited the highest prevalence of infection, at 11.11% (1/9), while *B*. *s*. *siamensis* displayed a prevalence of 1.39% (9/646). The greatest percentage of cercarial infection was detected in the snails collected from Songkhla Province (17.66%), followed by those collected from Nakhon Nayok (15.15%), Tak (14.29%), and Ayutthaya (0.48%) Provinces (Fig 1 and Table 1).

### Morphology of cercariae

**Fig 2:**
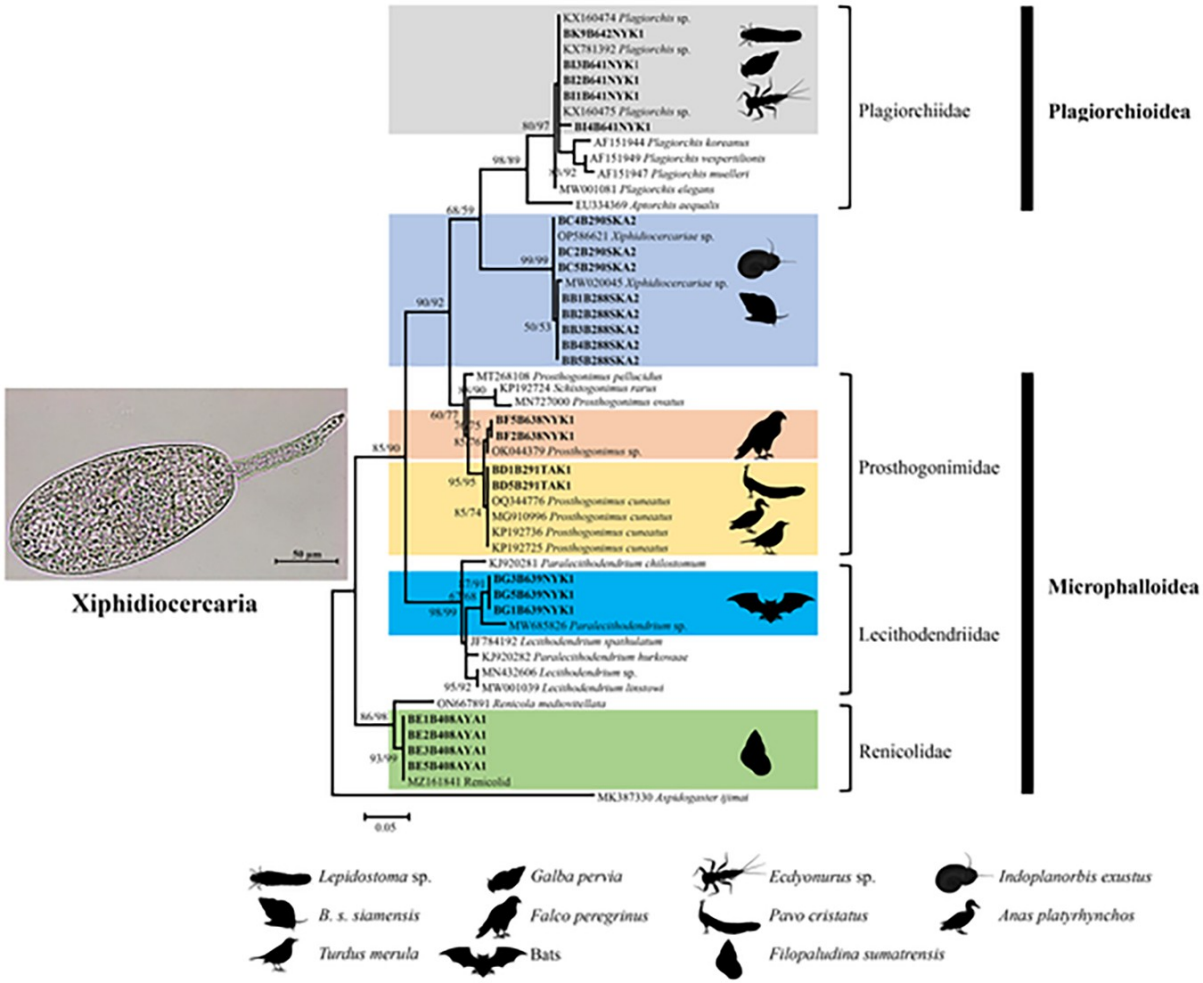
Maximum likelihood phylogenetic tree based on the ITS2 sequences of the xiphidiocercariae obtained from *B*. *s*. *siamensis* and *H*. *nassa* in Thailand, along with other sequences obtained from GenBank. Bootstrap values (≥50%) for ML (left) and NJ (right) analyses are indicated at branch nodes. Bold letters denote sequences obtained in this study. The black silhouettes represent the hosts of the trematode according to molecular data from GenBank. *Aspidogaster ijimai* was used as the outgroup.

Based on morphological characteristics, the cercariae found in this study were identified as xiphidiocercariae. They have a small, oval-elongated body measuring 170–175 μm in length and 80–90 μm in width. The oral sucker, round and located at the anterior end, features a distinctive stylet at its center, measuring 12–15 μm in length, a defining characteristic of this type. The ventral sucker, situated in the middle of the body, is globular and smaller than the oral sucker. The tail is tapered and shorter than the body, measuring 75–96 μm in length (Fig 2).

### Molecular analysis of cercariae

ITS2 was successfully amplified and sequenced from 24 xiphidiocercariae samples from 8 out of the 10 infected bithyniid snails. However, sequence amplification failed for cercariae from two *B*. *s*. *siamensis* strains from the provinces of Songkhla and Nakhon Nayok due to poor DNA quality. The ITS2 sequences (309–339 bp) of these xiphidiocercariae (GenBank accession nos. PP094634-PP094657) were identified as belonging to *Xiphidiocercariae* sp., *Prosthogonimus* sp., *Paralecithodendrium* sp., *Plagiorchis* sp., and renicolid sequences. The detailed results from the BLASTn analysis of the cercariae samples are shown in Table 3.

**Table 3:** Results from the BLASTn analysis based on ITS2 sequences of xiphidiocercariae samples.

Sequence ID	Location	No. of cercariae	Compared trematodes	GenBank Accession number	Identity (%)
BE1B408AYA1	Ayutthaya	1	Renicolid	MZ161841	100
BE2B408AYA1	Ayutthaya	1	Renicolid	MZ161841	100
BE3B408AYA1	Ayutthaya	10	Renicolid	MZ161841	100
BE5B408AYA1	Ayutthaya	5	Renicolid	MZ161841	100
BF2B638NYK1	Nakhon Nayok	1	*Prosthogonimu*s sp.	OK044379	99.35
BF5B638NYK1	Nakhon Nayok	1	*Prosthogonimu*s sp.	OK044379	99.68
BG1B639NYK1	Nakhon Nayok	1	*Paralecithodendrium* sp.	MW685826	93.31
BG3B639NYK1	Nakhon Nayok	1	*Paralecithodendrium* sp.	MW685826	92.98
BG5B639NYK1	Nakhon Nayok	5	*Paralecithodendrium* sp.	MW685826	93.31
BI1B641NYK1	Nakhon Nayok	1	*Plagiorchis* sp.	KX781392	99.70
BI2B641NYK1	Nakhon Nayok	1	*Plagiorchis* sp.	KX781392	100
BI3B641NYK1	Nakhon Nayok	3	*Plagiorchis* sp.	KX781392	100
BI4B641NYK1	Nakhon Nayok	3	*Plagiorchis* sp.	KX781392	98.82
BK9B642NYK1	Nakhon Nayok	5	*Plagiorchis* sp.	KX781392	100
BB1B288SKA2	Songkhla	1	*Xiphidiocercariae* sp.	MW020045	99.67
BB2B288SKA2	Songkhla	7	*Xiphidiocercariae* sp.	MW020045	99.33
BB3B288SKA2	Songkhla	2	*Xiphidiocercariae* sp.	MW020045	99.67
BB4B288SKA2	Songkhla	4	*Xiphidiocercariae* sp.	MW020045	99.67
BB5B288SKA2	Songkhla	4	*Xiphidiocercariae* sp.	MW020045	99.67
BC2B290SKA2	Songkhla	1	*Xiphidiocercariae* sp.	OP586621	100
BC4B290SKA2	Songkhla	2	*Xiphidiocercariae* sp.	OP586621	100
BC5B290SKA2	Songkhla	3	*Xiphidiocercariae* sp.	OP586621	100
BD1B291TAK1	Tak	1	*Prosthogonimus cuneatus*	MG910996	99.68
BD5B291TAK1	Tak	5	*Prosthogonimus cuneatus*	MG910996	100

The ML phylogenetic tree constructed from the ITS2 xiphidiocercariae sequences, comprising 24 sequences from this study and 26 sequences downloaded from GenBank (Table 4), revealed the presence of four different families within two superfamilies (Plagiorchioidea and Microphalloidea) of digenean trematodes. Two sequences (BD1B291TAK1 and BD5B291TAK1) of xiphidiocercariae released from *H*. *nassa* in Tak Province were closely related to *Prosthogonimus cuneatus*, a parasite within the family Prosthogonimidae known to infect Indian peafowl (*Pavo cristatus*), wild ducks (*Anas platyrhynchos*), and blackbirds (*Turdus merula*), exhibiting a genetic divergence of 0.32%. Moreover, five sequences (BI1B641NYK1-BI4B641NYK1 and BK9B642NYK1) of Xiphidiocercariae released from *B*. *s*. *siamensis* in Nakhon Nayok Province belonged to a clade of trematodes within the family Plagiorchiidae. These sequences closely clustered with those of *Plagiorchis* sp. found in the caddisfly (*Lepidostoma* sp.), snail (*Galba pervia*), and mayfly (*Ecdyonurus* sp.), indicating a genetic divergence of 0.29%. Furthermore, two sequences (BF2B638NYK and BF5B638NYK1) and three sequences (BG1B639NYK1, BG3B639NYK1, and BG5B639NYK1) from the same province were grouped with *Prosthogonimus* sp., which infects the bird *Falco peregrinus*, and *Paralecithodendrium* sp. in bats from the families Prosthogonimidae and Lecithodendriidae, respectively, showing genetic divergences of 0.32% and 10.09%, respectively. Four sequences (BE1B408AYA1-BE3B408AYA1 and BE5B408AYA1) from Ayutthaya Province closely matched the renicolid trematodes in the family Renicolidae, infecting the snail *Filopaludina sumatrensis*, with no genetic divergence (0.0%). However, eight samples (BB1B288SKA2-BB5B288SKA2, BC2B290SKA2, BC4B290SKA2, and BC5B290SKA2) from Songkhla Province clearly clustered as *Xiphidiocercariae* sp., which were isolated from snails *Indoplanorbis exustus* and *B*. *s*. *siamensis*. These samples were closely clustered between the families Plagiorchiidae and Prosthogonimidae, with a genetic divergence of 0.36% between these two identical species (Fig 2).

**Table 4:** ITS2 sequence data of cercariae obtained in this study and related trematodes acquired from GenBank database for the phylogenetic analyses.

Family	Trematode species or Sequence ID	Accession no.	Host	Location	Reference
Plagiorchiidae	*Plagiorchis* sp.	KX160474	*Lepidostoma* sp.	Germany	[41]
	*Plagiorchis* sp.	KX781392	*Galba pervia*	China	Ren et al. (unpublished)
	*Plagiorchis* sp.	KX160475	*Ecdyonurus* sp.	Germany	[41]
	*Plagiorchis koreanus*	AF151944	*Pipistrellus kuhli*	Ukraine	[42]
	*Plagiorchis vespertilionis*	AF151949	*Myotis daubentoni*	Ukraine	[42]
	*Plagiorchis muelleri*	AF151947	*Eptesicus serotinus*	Ukraine	[42]
	*Plagiorchis elegans*	MW001081	*Lymnaea stagnalis*	Denmark	[43]
	*Aptorchis aequalis*	EU334369	*Emydura kreffti*	Australia	[44]
	BI1B641NYK1	PP094653	*B*. *s*. *siamensis*	Thailand	in this study
	BI2B641NYK1	PP094654	*B*. *s*. *siamensis*	Thailand	in this study
	BI3B641NYK1	PP094655	*B*. *s*. *siamensis*	Thailand	in this study
	BI4B641NYK1	PP094656	*B*. *s*. *siamensis*	Thailand	in this study
	BK9B642NYK1	PP094657	*B*. *s*. *siamensis*	Thailand	in this study
Undefined taxa	*Xiphidiocercariae* sp.	OP586621	*Indoplanorbis exustus*	Thailand	[45]
	*Xiphidiocercariae* sp.	MW020045	*B*. *s*. *siamensis*	Thailand	[33]
	BB1B288SKA2	PP094634	*B*. *s*. *siamensis*	Thailand	in this study
	BB2B288SKA2	PP094635	*B*. *s*. *siamensis*	Thailand	in this study
	BB3B288SKA2	PP094636	*B*. *s*. *siamensis*	Thailand	in this study
	BB4B288SKA2	PP094637	*B*. *s*. *siamensis*	Thailand	in this study
	BB5B288SKA2	PP094638	*B*. *s*. *siamensis*	Thailand	in this study
	BC2B290SKA2	PP094639	*B*. *s*. *siamensis*	Thailand	in this study
	BC4B290SKA2	PP094640	*B*. *s*. *siamensis*	Thailand	in this study
	BC5B290SKA2	PP094641	*B*. *s*. *siamensis*	Thailand	in this study
Prosthogonimidae	*Prosthogonimus pellucidus*	MT268108	freshwater snails	Vietnam	[46]
	*Schistogonimus rarus*	KP192724	*Anas clypeata*	Czech Republic	[47]
	*Prosthogonimus ovatus*	MN727000	*Bithynia tentaculata*	Germany	[48]
	*Prosthogonimus* sp.	OK044379	*Falco peregrinus*	United Arab Emirates	[49]
	*Prosthogonimus cuneatus*	OQ344776	*Pavo cristatus*	India	[50]
	*Prosthogonimus cuneatus*	MG910996	*Anas platyrhynchos*	Vietnam	Huynh et al. (unpublished)
	*Prosthogonimus cuneatus*	KP192736	*Turdus merula*	Czech Republic	[47]
	*Prosthogonimus cuneatus*	KP192725	*Anas platyrhynchos*	Czech Republic	[47]
	BF2B638NYK1	PP094644	*B*. *s*. *siamensis*	Thailand	in this study
	BF5B638NYK1	PP094645	*B*. *s*. *siamensis*	Thailand	in this study
	BD1B291TAK1	PP094642	*H*. *nassa*	Thailand	in this study
	BD5B291TAK1	PP094643	*H*. *nassa*	Thailand	in this study
Lecithodendriidae	*Paralecithodendrium chilostomum*	KJ920281	*Nyctalus noctula*	Ukraine	[51]
	*Paralecithodendrium* sp.	MW685826	bats	Egypt	Abuelmaged et al. (unpublished)
	*Paralecithodendrium hurkovaae*	KJ920282	*Myotis daubentoni*	Ukraine	[51]
	*Lecithodendrium spathulatum*	JF784192	*Pipistrellus pipistrellus*	England	[52]
	*Lecithodendrium* sp.	MN432606	*Filopaludina sumatrensis*	Thailand	[53]
	*Lecithodendrium linstowi*	MW001039	*Stagnicola palustris*	Denmark	[43]
	BG1B639NYK1	PP094650	*B*. *s*. *siamensis*	Thailand	in this study
	BG3B639NYK1	PP094651	*B*. *s*. *siamensis*	Thailand	in this study
	BG5B639NYK1	PP094652	*B*. *s*. *siamensis*	Thailand	in this study
Renicolidae	*Renicola mediovitellata*	ON667891	*Nucella lapillus*	Iceland	[54]
	Renicolid	MZ161841	*Filopaludina sumatrensis*	Thailand	[25]
	BE1B408AYA1	PP094646	*B*. *s*. *siamensis*	Thailand	in this study
	BE2B408AYA1	PP094647	*B*. *s*. *siamensis*	Thailand	in this study
	BE3B408AYA1	PP094648	*B*. *s*. *siamensis*	Thailand	in this study
	BE5B408AYA1	PP094649	*B*. *s*. *siamensis*	Thailand	in this study
Aspidogastridae	*Aspidogaster ijimai* (outgroup)	MK387330	*Cyprinus carpio*	Japan	[55]

### Molecular identification of snails

The COI and 16S rDNA sequences of bithyniid snails infected with cercariae were analyzed via PCR amplification and sequencing. Eight out of the ten bithyniid snails infected with cercariae were successfully amplified and sequenced. Sequence amplification failed for two snails from Ayutthaya and Nakhon Nayok provinces due to poor DNA quality. The molecular identification of bithyniid snails based on the COI and 16S rDNA genes was consistent with the morphological identification. Seven sequences (GenBank accession nos. PP094598-PP094604) of 578 bp of the COI gene were obtained from *B*. *s*. *siamensis*. BLASTn results showed 100% similarity to *B*. *s*. *siamensis* from Thailand (GenBank accession nos. KY118649, KY118672, MW832442, and KY118653), while one sequence (GenBank accession no. PP094614) from *H*. *nassa* displayed 100% similarity with *H*. *nassa* from Thailand (GenBank accession no. MK640188). Additionally, the 16S rDNA sequences (367–369 bp) from 7 samples (GenBank accession nos. PP094615-PP094621) of *B*. *s*. *siamensis* in the present study showed 98.62–100% similarity to those of *B*. *s*. *siamensis* from Thailand (GenBank accession nos. MW305394 and MW305395). Moreover, one sample (GenBank accession no. PP094628) of *H*. *nassa* exhibited 100% homology to *H*. *nassa* from Thailand (GenBank accession no. MK629223).

### Phylogenetic analysis of snails

**Fig 3:**
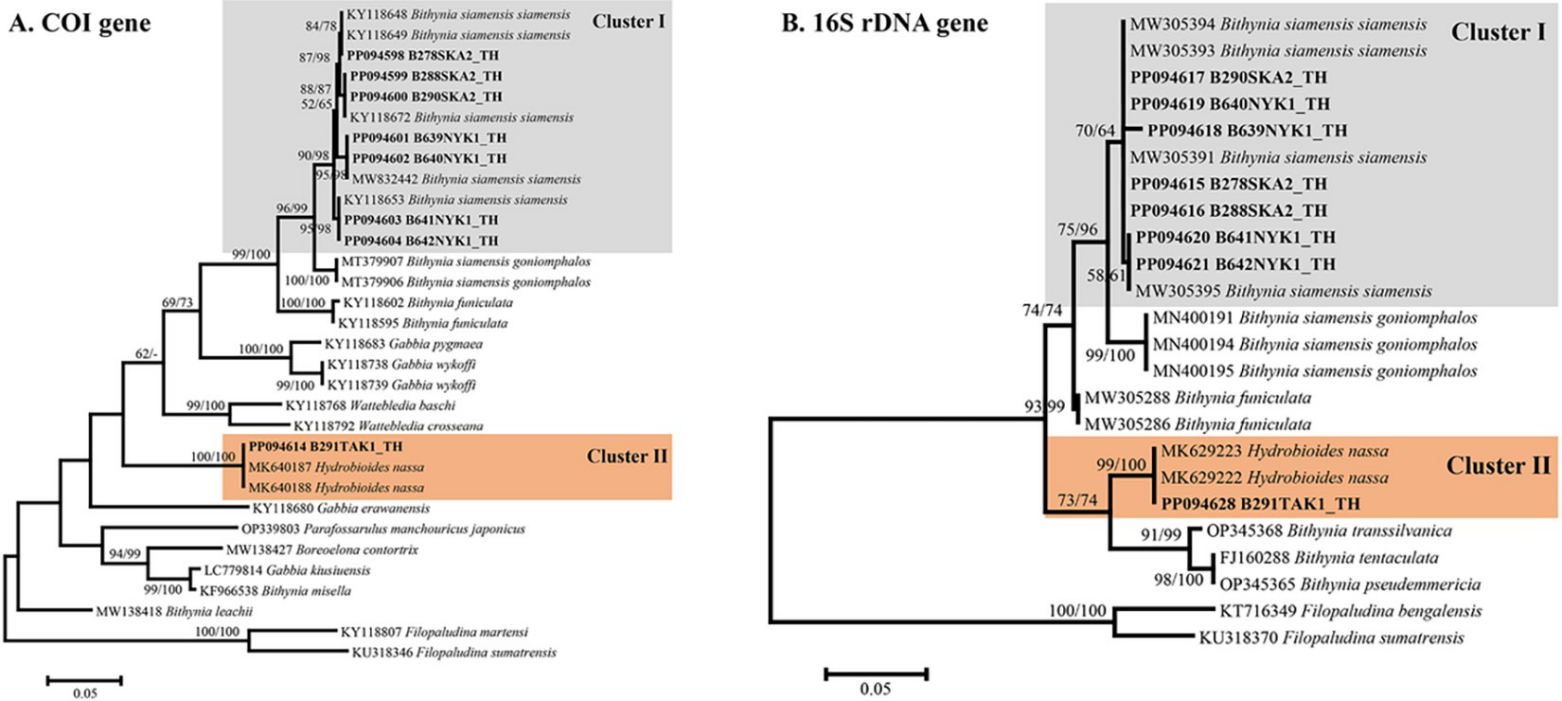
Maximum likelihood phylogenetic tree based on COI (A) and 16S rDNA (B) sequences of bithyniid snails in Thailand, along with other sequences obtained from GenBank. Bootstrap values (≥50%) for ML (left) and NJ (right) analyses are indicated at branch nodes. Bold letters denote sequences obtained in this study. *Filopaludina martensi*, *F*. *sumatrensis*, and *F*. *bengalensis* were used as the outgroup.

Phylogenetic analyses of bithyniid snails based on COI and 16S rDNA revealed congruent clustering results. The ML phylogenetic tree of the COI (578 bp) and 16S rDNA (367–369 bp) sequences showed that our *B*. *s*. *siamensis* obtained from Songkhla and Nakhon Nayok provinces clustered with *B*. *s*. *siamensis* from Thailand, while *H*. *nassa* sequences from Tak Province grouped together with *H*. *nassa* sequences from Thailand (Fig 3).

## Discussion

Bithyniid snails are common freshwater gastropods in Thailand and are found in various types of water bodies [20]. Overall, our study revealed that bithyniid snails were prevalent in most paddy fields. Morphological identification revealed three species, *B*. *funiculata*, *B*. *s*. *siamensis*, and *H*. *nassa*, with *B*. *s*. *siamensis* being the most abundant, followed by *B*. *funiculata* and *H*. *nassa*. These findings are consistent with previous research [1,19,20,56], which reported the wide distribution of *B*. *s*. *siamensis* across five regions (central, northern, northeastern, western, and southern) of Thailand. However, our study revealed a new occurrence of *B*. *s*. *siamensis* in the eastern region, which differs from previous reports.

In this study, we performed molecular analysis of mitochondrial COI and 16S rDNA sequences to identify the species of bithyniid snails harboring trematode cercariae in Thailand. In our study, *B*. *s*. *siamensis* was confirmed to have 100% identity for the COI sequence and 98.62–100% identity for the 16S rDNA sequence after BLASTn searching. Similarly, *H*. *nassa* showed 100% identity with *H*. *nassa* sequences from the GenBank database. Consistent with the phylogenetic analysis, the ML phylogenetic tree of the COI and 16S rDNA sequences obtained in our study showed that *B*. *s*. *siamensis* and *H*. *nassa* clustered with *B*. *s*. *siamensis* and *H*. *nassa* from Thailand, respectively. Our findings align with those of Bunchom et al. (2021) [18,20], who described the molecular identification of these two snail species in Thailand through the analysis of different mitochondrial COI and 16S rDNA markers. Furthermore, we observed that the morphologies of *B*. *s*. *siamensis* and *H*. *nassa* are very similar, potentially causing taxonomic confusion when traditional morphological characters are used for identification and classification [18]. Therefore, our results indicate that COI and 16S rDNA markers are effective for confirming and differentiating between *B*. *s*. *siamensis* and *H*. *nassa* [18–20].

In our study, we found that the overall rate of cercarial infection in bithyniid snails was 1.45%. This rate was lower than the 3.15% reported by Kulsantiwong et al. (2015) [21] for bithyniid snails in Thailand. Variations in cercarial infection rates in snails can be influenced by numerous factors, such as differences in collection times, seasons, locality, rainfall, temperature, water quality, availability of infected definitive hosts, type and number of parasites and intermediate hosts, and snail population density [57–60]. The low infection rate in our study may be due to reduced parasite presence, which limits contact between snails and miracidia [27,61]. Additionally, the limited diversity of secondary intermediate hosts and definitive hosts at the sampling sites could also contribute to the reduced infection rate [27,62]. Moreover, decreased human activities such as open-field defecation, farming, and livestock grazing have been associated with lower trematode infection rates in snails [27,63].

In the present study, only xiphidiocercariae were detected in *H*. *nassa* and *B*. *s*. *siamensis*. The morphology of the xiphidiocercariae observed was consistent with the characteristics reported for xiphidiocercariae collected from *B*. *s*. *siamensis* in Bangkok Province [33]. Previous research also reported xiphidiocercariae infection in *H*. *nassa* in Chiang Rai Province, Thailand [17]. Our results indicate new areas of xiphidiocercariae distribution in *H*. *nassa* in Tak Province and in *B*. *s*. *siamensis* in Ayutthaya and Nakhon Nayok Provinces. Xiphidiocercariae were the predominant type of cercaria found in *H*. *nassa* and *B*. *s*. *siamensis* in this investigation, consistent with previous studies indicating that xiphidiocercariae are the most frequently observed type in other bithyniid snails [21,22,24]. This cercaria type typically requires birds, reptiles, or mammals as definitive hosts to mature into adult intestinal flukes [41,42,47,64]. The high prevalence of xiphidiocercariae observed in this study may be attributed to the coexistence of definitive and intermediate hosts within the same ecosystem, which facilitates the completion of the parasite’s life cycle [27]. Another aspect to consider is that *H*. *nassa* and *B*. *s*. *siamensis* may be susceptible to xiphidiocercariae infection. This phenomenon was previously reported in the snail *B*. *s*. *siamensis* [30], indicating that *B*. *s*. *siamensis* is more susceptible to *O*. *viverrini* infection than is *B*. *funiculata*. Schmid-Hempel and Stauffer (1998) [65] highlighted that the susceptibility of hosts to parasites can increase when genetic variation within the host population decreases. Lee et al. (1995) [66] investigated trematode (*Fasciola hepatica*) infection in *Orientogalba viridis* and reported that this snail is highly susceptible to infection at the miracidia stage across various growth stages. These findings indicate a well-established relationship between the parasite and its snail host, suggesting that *H*. *nassa* and *B*. *s*. *siamensis* are susceptible to xiphidiocercariae infection. However, there is limited research investigating the dynamics of the relationship between bithyniid snails and xiphidiocercariae. Thus, the susceptibility of *H*. *nassa* and *B*. *s*. *siamensis* to xiphidiocercariae infection remains uncertain. Further studies are necessary to reach definitive conclusions on this matter.

The molecular identification of the cercariae was performed using ITS2 sequences, which were analyzed to construct a phylogenetic tree. The ML phylogenetic tree based on the xiphidiocercariae sequences classified them into four different families: Plagiorchiidae (including *Plagiorchis* sp.), Prosthogonimidae (*Prosthogonimus* sp. and *Prosthogonimus cuneatus*), Lecithodendriidae (*Paralecithodendrium* sp.), and Renicolidae (Renicolid). This cercarial morphotype has been shown to produce a wide variety of trematode species, a finding consistent with previous studies [33]. In this study, we report for the first time the identification of *Plagiorchis* sp., *Prosthogonimus* sp., *Paralecithodendrium* sp., and renicolid in *B*. *s*. *siamensis*, as well as *Prosthogonimus cuneatus* in *H*. *nassa* from Thailand. Among these species, *Plagiorchis* and *Paralecithodendrium* are known to cause diseases in humans [67,68], while the other species have veterinary significance [54,64].

Many species of *Plagiorchis* species are known to parasitize the intestines of vertebrates [69]. They have also been identified as potential pathogens in humans, causing plagiorchiasis, with 12 recorded cases mainly in Asia [67,69–71]. The source of *Plagiorchis* infection in humans is presumed to be freshwater fish, freshwater snails, and aquatic arthropods [72]. In Thailand, adult *Plagiorchis* sp. were first identified in four opisthorchiasis patients following praziquantel treatment between 1980 and 1985 in the northeast region [68]. Moreover, *Paralecithodendrium* species are parasitic trematodes found in fish, amphibians, birds, and mammals, predominantly bats [73]. Specifically, in Thailand, cercariae of this parasite have been reported in *B*. *s*. *goniomphalos* and *Filopaludina martensi martensi* snails [24,74]. Additionally, the infective metacercariae stage has been detected in insects of the order Odonata in the northeastern region of Thailand [75]. Notably, *Paralecithodendrium molenkampi* is a well-known species infecting human in Thailand [67]. In contrast, *Prosthogonimus* species are significant pathogenic parasites in wild birds and poultry, leading to mortality and defective egg formation [64]. These parasites utilize dragonfly nymphs or dragonflies as their second intermediate hosts [47]. Previous research has reported that *B*. *s*. *goniomphalos* is the first intermediate host of *Prosthogonimus* in Thailand [24]. Despite the absence of reported infections in wild birds or poultry in Thailand, the presence of *Prosthogonimus* poses a potential threat to these populations. Renicolid trematodes are commonly known as a group of parasites inhabiting the kidneys and ureters of aquatic birds and exerting a strong pathogenic effect on their hosts. The metacercariae of these parasites develop in second intermediate host freshwater snails and fishes [54]. However, data on their distribution and infectivity in Thailand are scarce, with reports of metacercariae found in *F*. *martensi*, *F*. *sumatrensis*, and *Idiopoma umbilicata* snails in Bangkok, Thailand [25].

## Conclusion

In conclusion, our study documents the presence and variety of trematode larvae in bithyniid snails across Thailand. Our findings revealed that three species/subspecies of bithyniid snails, including *B*. *funiculata*, *B*. *s*. *siamensis*, and *H*. *nassa*, were distributed variably across five regions. Two species/subspecies of snails within this family act as intermediate hosts for significant medical and veterinary trematodes. This study also provides new information on the intermediate hosts of two trematode species affecting human public health and three species of veterinary significance. Given the public health risk posed by trematode parasites in bithyniid snails, particularly the presence of the zoonotic trematode species *Plagiorchis* sp. and *Paralecithodendrium* sp., these findings underscore the important role of these snails as intermediate hosts for trematode cercariae in Thailand.
